# Coordinated Regulation of SIV Replication and Immune Responses in the CNS

**DOI:** 10.1371/journal.pone.0008129

**Published:** 2009-12-17

**Authors:** Kenneth W. Witwer, Lucio Gama, Ming Li, Christopher M. Bartizal, Suzanne E. Queen, John J. Varrone, Angela K. Brice, David R. Graham, Patrick M. Tarwater, Joseph L. Mankowski, M. Christine Zink, Janice E. Clements

**Affiliations:** 1 Department of Molecular and Comparative Pathobiology, Johns Hopkins University School of Medicine, Baltimore, Maryland, United States of America; 2 Department of Pathology, Johns Hopkins University School of Medicine, Baltimore, Maryland, United States of America; 3 Department of Neurology, Johns Hopkins University School of Medicine, Baltimore, Maryland, United States of America; 4 Department of Medicine, Johns Hopkins University School of Medicine, Baltimore, Maryland, United States of America; 5 Department of Biomedical Sciences, Texas Tech University Health Sciences Center, El Paso,Texas, United States of America; University of California San Francisco, United States of America

## Abstract

Central nervous system (CNS) invasion during acute-stage HIV-infection has been demonstrated in a small number of individuals, but there is no evidence of neurological impairment at this stage and virus infection in brain appears to be controlled until late-stage disease. Using our reproducible SIV macaque model to examine the earliest stages of infection in the CNS, we identified immune responses that differentially regulate inflammation and virus replication in the brain compared to the peripheral blood and lymphoid tissues. SIV replication in brain macrophages and in brain of SIV-infected macaques was detected at 4 days post-inoculation (p.i.). This was accompanied by upregulation of innate immune responses, including IFNβ, IFNβ-induced gene MxA mRNA, and TNFα. Additionally, IL-10, the chemokine CCL2, and activation markers in macrophages, endothelial cells, and astrocytes were all increased in the brain at four days p.i. We observed synchronous control of virus replication, cytokine mRNA levels and inflammatory markers (MHC Class II, CD68 and GFAP) by 14 days p.i.; however, control failure was followed by development of CNS lesions in the brain. SIV infection was accompanied by induction of the dominant-negative isoform of C/EBPβ, which regulates SIV, CCL2, and IL6 transcription, as well as inflammatory responses in macrophages and astrocytes. This synchronous response in the CNS is in part due to the effect of the C/EBPβ on virus replication and cytokine expression in macrophage-lineage cells in contrast to CD4+ lymphocytes in peripheral blood and lymphoid tissues. Thus, we have identified a crucial period in the brain when virus replication and inflammation are controlled. As in HIV-infected individuals, though, this control is not sustained in the brain. Our results suggest that intervention with antiretroviral drugs or anti-inflammatory therapeutics with CNS penetration would sustain early control. These studies further suggest that interventions should target HIV-infected individuals with increased CCL2 levels or HIV RNA in the CNS.

## Introduction

HIV infection of the brain is thought to occur during acute infection based on a limited number of case reports and studies [1]–[3]. Because the earliest stage of infection cannot be studied in humans, it is not clear whether acute infection in the central nervous system (CNS) elicits inflammatory responses similar to those that are observed during late-stage infection in the brain. It has been postulated that HIV infection is cleared from the brain after acute infection, and that CNS deficits occur because of re-entry of virus into the CNS during late-stage disease—when there is high viral load in peripheral blood and immune impairment [4], [5]. This is particularly relevant in the current HAART-era, when patients frequently maintain suppression of virus replication in the peripheral blood. Despite this control of patient virus replication, HIV-associated neurocognitive disorders are prevalent and increasing [6]–[8]. Thus, it is essential to understand the innate mechanisms in the brain that may naturally suppress both virus replication and the accompanying inflammatory responses that are linked to eventual loss of neuronal function and neuronal apoptosis in the CNS.

The regulation of virus replication in the brain and the peripheral blood may be different because productively infected cells in brain are of macrophage-lineage, in contrast to CD4+ lymphocyte in the peripheral blood and immune tissues [9]–[13]. Our reproducible SIV macaque model of HIV/AIDS and CNS disease provides an opportunity to examine the brain and periphery during specific time points during acute and early infection. We previously have characterized innate immune response in the brain that regulated the transcriptional regulation of virus in infected macrophages *in vitro* and *in vivo* during acute infection.

Our earlier studies in the SIV macaque model demonstrated that there is virus replication in the brain during acute infection. After acute infection, virus is not completely cleared from the brain despite reduction in viral replication. SIV DNA levels remain constant in the brain from acute- to late-stage disease; however, there is a shift in transcriptionally active to inactive SIV DNA during the asymptomatic period [12], [14], [15]. Induction of innate immune responses in brain macrophages—the predominant productively-infected cells in the brain—can suppress SIV gene expression in these cells, resulting in cerebrospinal fluid (CSF) and brain viral load reductions. We have further demonstrated that induction of IFNβ, the first type I IFN to be produced in response to viral infections, reduces SIV replication *in vitro* in primary macaque macrophages by a transcriptional mechanism. Additionally, acute infection IFNβ induction in the brain correlates with repression of SIV transcription in macrophages; this correlation is also observed with the IFNβ downstream transcriptional regulatory pathway protein C/EBPβ, a member of the CCAAT/enhancer binding protein family of transcription factors in macrophages [14]–[26]. In addition to controlling virus infection, IFNβ-induced genes—particularly C/EBPβ—regulate inflammatory gene cascades, limiting inflammation and damage in tissues [26]–[31].

Understanding the regulation of inflammatory pathways in the brain during infection and the molecular events linked to control of inflammation is critical to preventing the development of HIV encephalitis and cognitive changes observed in HIV-infected individuals. HIV-1 and SIV infection upregulate the inflammatory cytokines IL1β, TNFα and IL-6 in brain in macrophages, as well as the chemokine CCL2 in macrophages and astrocytes [32]–[39]; this induction, along with the induction of type I IFNs, promotes antiviral responses. However, these cytokines, including IFNβ and C/EBPβ, also prevent inflammatory responses from continuing unchecked [26]–[31]. IFNβ regulates the transcription factor C/EBPβ, which is critical to the transcriptional regulation of the proinflammatory genes IL-6, CCL2 and TNFα [29], [40], [41]. Previous studies in our model demonstrated that increased levels of C/EBPβ in macrophages and the brain led to the decreases in RNA transcription and SIV promotor histone acetylation [14], [15]. The dominant-negative form of C/EBPβ also may be important for regulation of proinflammatory genes in the HIV-1- or SIV-infected brain.

Using our SIV macaque model of HIV/AIDS and CNS disease, we demonstrate in this study that infection of the brain occurs during the earliest phase of acute infection. By 4 days post-inoculation (p.i.), SIV replication is detected in CD14+ macrophages in the brain and infection is accompanied by a widespread innate immune response. Further, there is coordinated induction of TNFα, IL10 and CCL2 in the brain during acute infection. Following acute infection (4–10 days p.i.), there is a decrease in the levels of proinflammatory cytokines in the brain, accompanied by a reduction in inflammatory markers in macrophages, endothelial cells and astrocytes in brain. This downregulation of cytokines and SIV in the brain is not sustained; increased expression can occur macaques with development of CNS disease by 42 days p.i.

## Results

In previous studies we examined SIV virus load, CCL2 and IL6 protein in CSF from infected macaques [9], [36], [37], [42]–[44]. In this study, we quantified virus replication, proinflammatory cytokines and innate immune responses directly in the brain of SIV-infected macaques at multiple times during acute and early stages of infection to correlate them with longitudinal disease progression. In addition, SIV-infected macaques were euthanized at 42 days p.i. to examine cytokine and innate immune responses that are present during resurgence of virus replication.

### SIV Infection and Innate Immune Responses in Brain at 4 Days P.I

**Figure 1:**
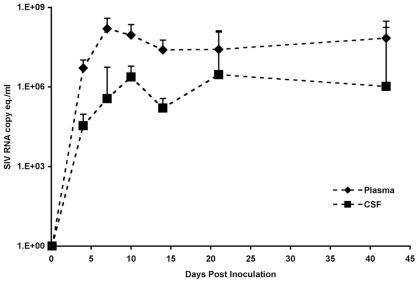
Quantitation of SIV virion RNA in plasma and CSF of SIV-infected macaques. SIV RNA was isolated from 140 µl of plasma and CSF collected at terminal time points from uninfected and SIV-infected macaques sacrificed at 4, 7, 10, 14, 21, or 42 days p.i. SIV RNA copy equivalents were determined by quantitative RT-PCR, and the means (diamonds for plasma and squares for CSF) and standard deviation for each experimental group are indicated.

Based on our previous studies, SIV viral load is detectable at 7 days p.i. in the plasma and CSF of infected macaques, while SIV mRNA in the brain is observed at 7 and 10 day p.i. [9], [36], [37], [42], [43]. In this study, we examined the brain of SIV-infected macaques at 4 days p.i. to determine when virus infection in the brain occurs compared to the peripheral blood. There was significant virus in the peripheral blood (median of 5.2×10^6^ SIV RNA copy eq./ml plasma in 6 SIV-infected macaques) and in CSF (median of 3.5×10^4^ RNA copy eq./ml CSF) at 4 d p.i. at these early time points (Figure 1). SIV RNA also was detected in the brain (basal ganglia and the adjacent white matter and cerebral cortex) in 6 of 6 SIV-infected macaques at this early time point (Table 1). Since there is no evidence that there is breakdown of the blood brain barrier at or that virus is transported across the blood brain barrier these data strongly suggest that virus enters and replicates in the brain during the earliest period of acute infection in the peripheral blood.

**Table 1:** SIV Replication in Brain 4 Days P.I.

	SIV RNA Copy equivalents/ug RNA
Brain homogenate	191 (47–399)
CD14+ selected macrophages	9,390 (148–14,550)
CD11b+/CD14− selected macrophages	60 (<10–198)

To determine whether macrophages in the brain are infected at 4 days p.i., macrophages were isolated from brain of the SIV-infected macaques euthanized at 4 days p.i. (as previously described [45]). From the isolated brain macrophages, CD14+ macrophages were selected with CD14-antibody coated beads and the remaining unselected cells were then selected with CD11b-antibody coated beads. The CD14+ macrophages have recently entered the brain and are located in the perivascular region, while the CD11b+ (CD14−) macrophages represent the resident brain microglia in the brain parenchyma [46]–[50]. The CD14+ macrophages had significantly higher levels of SIV RNA than the brain homogenates from basal ganglia or parietal cortex (Table 1), while the CD11b+ population had low or no detectable SIV RNA. These data strongly suggest that the CD14+ macrophages that have recently entered the brain are the source of SIV replication during the earliest period of CNS infection and virus is not replicating to a significant level in microglia at this time.

Viral infection in the brain is accompanied by innate immune responses, in particular, the induction of mRNA for IFNβ and MxA [14], [15], [25]. Almost all cells express the IFNα/β receptor, thus, once the innate immune response is initiated by infection in the cell, soluble IFNβ binds to many cells inducing IFNβ and IFN-induced genes, such as MxA. The levels of IFNβ and MxA mRNAs were measured in brain homogenates and isolated macrophages from the brain of SIV-infected macaques euthanized at 4 days p.i. (Table 2). IFNβ mRNA was induced 1.8-fold in SIV-infected brain homogenate over uninfected levels; this induction was comparable to levels induced in SIV-infected macrophages *in vitro* [14], [25]. The increase in IFNβ mRNA has a multiplicative effect on the downstream MxA mRNA, which is induced >50 fold (Table 2), reflecting the induction of the innate immune responses in the brain at 4 days p.i. In comparison when the CD14+ macrophages were analyzed, there was a 38 fold increase in the induction of IFNβ mRNA and over a 800 fold increase in MxA RNA, reflecting the response to SIV replication in these cells as well as the paracrine response to IFNβ produced by the infected CD14+ macrophages. In contrast, the CD11b+ microglia that contain low levels of SIV RNA had a 200 fold increase in IFNβ mRNA and a 830 fold increase in MxA RNA, reflecting a paracrine response to the IFNβ produced in the infected CD14+ macrophages.

**Table 2:** Innate Immune Response in Brain 4 Days P.I.

	IFNβ mRNA*	MxA mRNA*
Brain homogenate	1.8	54.1
CD14+ selected macrophages	37.8	813.8
CD11b+/CD14− selected macrophages	213.0	831.9

*Fold increase compared to uninfected control levels in brain.

### SIV Replication in Peripheral Blood and Brain

To compare virus replication in peripheral blood, CSF, and brain, SIV RNA isolated from plasma and CSF was measured by quantitative RT-PCR at the terminal time point for each of the 55 SIV-infected macaques (Figure 1). There was a difference in the increase in virus during acute infection in plasma (4–7 days p.i.) compared to CSF (4–7 days p.i. and 4–10 days p.i.) as well as in the peak of levels of virus, in plasma the peak was at 7 days p.i. compared to 10 days p.i. in CSF. The difference in levels and peak of virus in the peripheral blood and CSF could be due to the delay in virus infected cells entering the brain as well as the different cells in the two compartments that support virus replication, predominantly, CD4+ lymphocytes in the peripheral blood compared to macrophages in the brain.

In both plasma and CSF, viral load decreased approximately 10-fold from the peak level at 14 days p.i. Decrease in the levels of virus occurred more rapidly in CSF (from 10–14 days p.i.) than in plasma (from 7–14 days p.i. and from 10–14 days p.i). The decrease in SIV replication in brain has been shown in our model to be due to the effects of IFNβ produced in response to infection that reduces SIV transcription in macrophages [9], [14] but does not have this effect in CD4+ lymphocytes. Further, the more rapid decline in SIV in CSF suggests that there are different mechanisms that control SIV replication in plasma versus CSF or brain.

**Figure 2:**
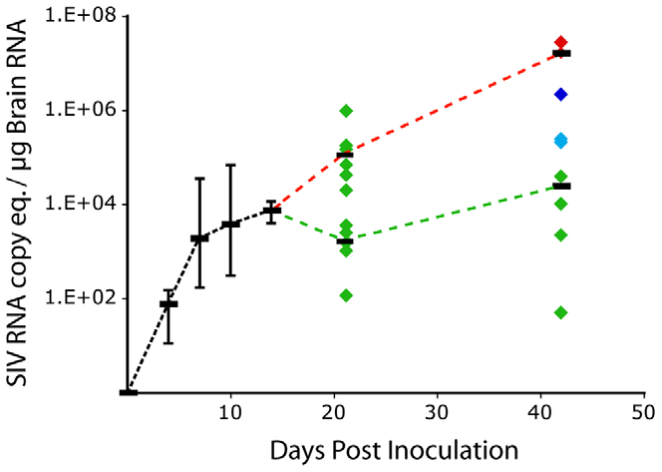
SIV RNA expression in brain of SIV-infected macaques. SIV RNA copies were quantitated by quantitative RT-PCR in RNA isolated from brain collected at each terminal time point from uninfected and SIV-infected macaques (4, 7, 10, 14, 21, or 42 days after inoculation). Medians (black bars) and the range (vertical bars) for each experimental group are indicated. The 21 day group (12 macaques) were split into two groups that had viral RNA levels above or below the median of the 14 day macaques; the median for these two groups are shown. For the 42 day group, each diamond represents one animal, color-coded according to CNS disease severity (red - severe; blue - moderate; turquoise - mild; green–none), and the black bars represent the medians for severe/moderate (red dotted line), and mild/none animals (green dotted line).

Median brain SIV RNA levels for six macaques showed a rapid increase between 4 and 10 days p.i., with very little change between 10 and 14 days p.i. (Figure 2). SIV RNA levels were more variable at 21 days p.i. The rapid increase in SIV RNA from 4–10 days p.i., followed by the small increase between 10 and14 days p.i., indicates control of SIV replication, which is reflected in the CSF as a 10-fold reduction in virus. At 42 days p.i., SIV RNA levels showed a wide range of virus replication: from levels below day 4–21 days p.i. to levels 100–1000–fold higher than 21 days p.i. Thus, in some of the SIV-infected macaques virus replication appears to have been controlled, while in others virus replication resurged to levels much higher than during acute and early infection. However, there was no difference in the level of virus in the plasma of the macaques that controlled SIV replication in the brain compared to those that did not. The level of SIV RNA in brain correlated with the severity of CNS lesions (r = 0.95; p<0.001) (Table 3) while there was no correlation between the level of virus load in the plasma and the severity of CNS lesions.

**Table 3:** Severity of CNS Lesions and SIV RNA in Brain at 42 days P.I.

Animal	Severity of CNS lesions	SIV RNA copy equiv./ug RNA Brain
PQw2	None	49
PLc2	None	2,169
PLi2	None	10,048
PWf2	None	38,600
PEe2	Mild	202,067
PGe2	Mild	238,300
PFc2	Moderate	2,104,000
PRd2	Severe	16,226,667
PLb2	Severe	26,876,667

### Innate Immune Responses in SIV-Infected Brain

**Figure 3:**
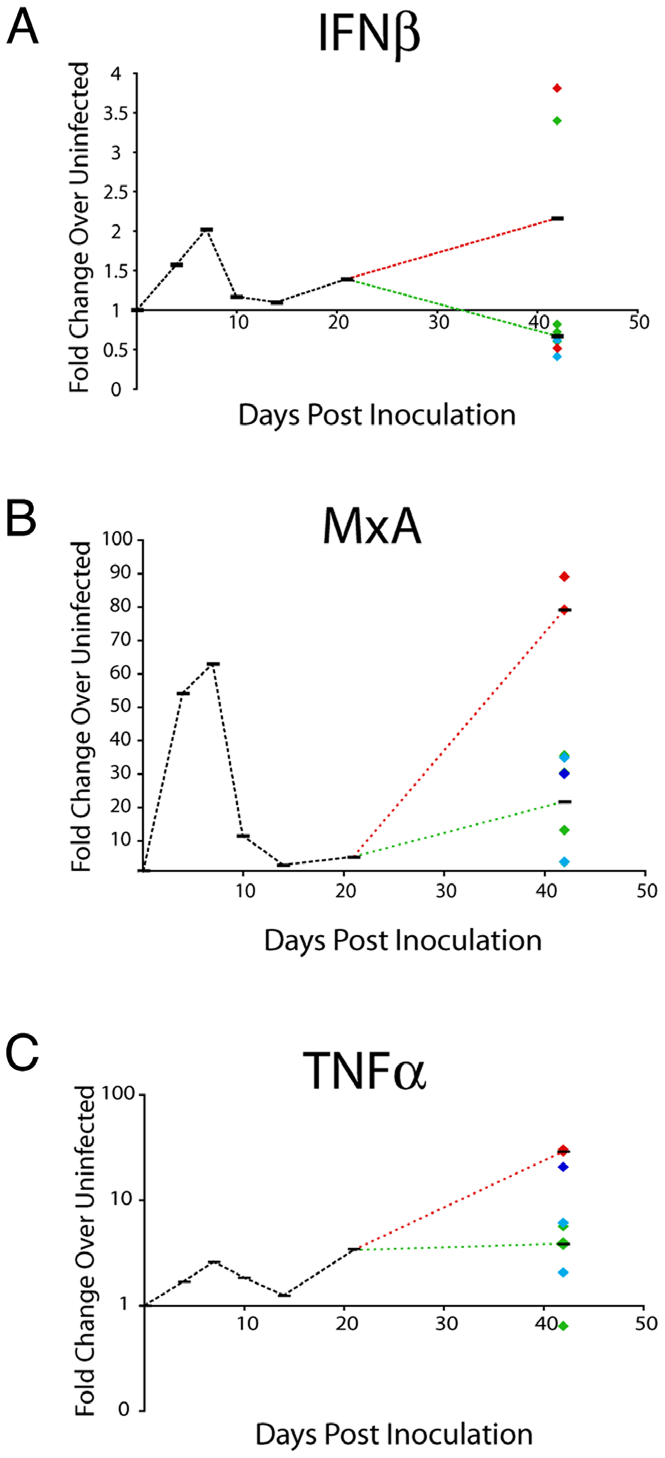
Quantitation of mRNA in brain of SIV-infected macaques. A) IFNβ; B) MxA; and C) TNFα mRNA was isolated from brain of uninfected and SIV-infected macaques at terminal time points (4, 7, 10, 14, 21, or 42 days after inoculation) and mRNA levels quantitated by quantitative RT-PCR. mRNA levels in the SIV-infected brain are represented as fold change over the average of three uninfected brain RNAs, calculated by ΔΔCt. Medians (black bars) for each experimental group are indicated. For the 42-day group, each diamond represents one animal, color-coded according to CNS disease severity (red-severe; blue-moderate; turquoise-mild; green-none), and the black bars represent the medians for severe/moderate (red dotted line), and mild/none animals (green dotted line). Outliers whose values were higher than 5 times the standard deviation for each group were excluded.

In these studies, IFNβ and MxA mRNA levels (as well as all of the cytokine RNA levels) were quantified by the ΔΔCT method, normalized to levels in uninfected control macaques, and reported as fold-change in RNA copies (Figure 3). IFNβ mRNA was induced 1.6- and 2.0-fold over levels in uninfected animals at 4 and 7 days p.i. respectively, and was reduced to comparable with uninfected animals at 10 and 14 days p.i. The IFNβ-induced gene MxA was induced 54- and 63-fold over uninfected levels at 4 and 7 days, respectively, in parallel with IFNβ induction; and at 10 and 14 days induction was reduced to 11- and 2.7-fold, respectively. IFNβ and MxA mRNA levels were elevated over uninfected control levels in the brain of 3/11 macaques euthanized at 21 days p.i. At 42 days p.i., IFNβ levels in the brains of 6/9 SIV-infected macaques were lower than in uninfected animals and in the remaining animals they were induced 3–4 fold over uninfected levels. There was no correlation with SIV RNA levels; however, MxA in the brain of SIV-infected macaques sacrificed at 42 days p.i also varied widely, but levels were correlated with SIV RNA levels in the macaques with severe and moderate CNS lesions (r = 0.90, p<0.001) (Table 3).

**Figure 4:**
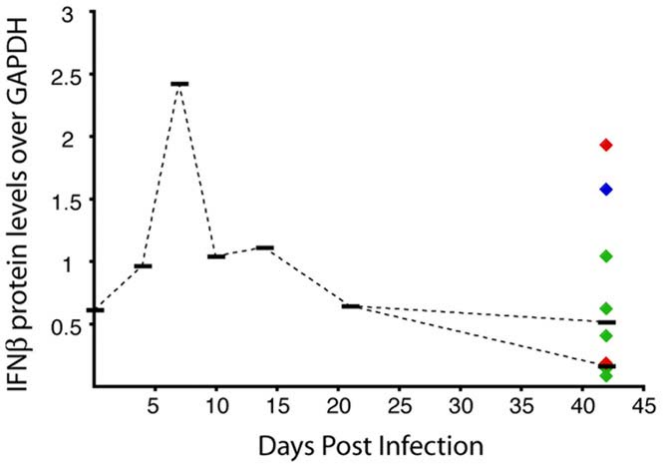
IFNβ protein levels in brain of SIV-infected macaques. Brain homogenates were made from brain from uninfected and SIV-infected macaques at terminal time points (4, 7, 10, 14, 21, or 42 days after inoculation) and IFNβ protein was quantitated by quantitative western analyses as described in Methods and Materials. Medians (black bars) for each experimental group are indicated. For the 42 day group, each diamond represents one animal, color-coded according to CNS disease severity (red-severe; blue-moderate; turquoise-mild; green-none), and the black bars represent the medians for severe/moderate (red dotted line), and mild/none animals (green dotted line). Protein band intensities were normalized to GAPDH.

IFNβ RNA is regulated by sequences in the 3′ UTR that mediate rapid turnover of the RNA [51]. To assess whether IFNβ protein levels in brain correlated directly with SIV RNA levels, IFNβ protein in brain was measured by quantitative Western analyses (Figure 4). IFNβ protein levels increased 1.5- and 4.0-fold compared to uninfected levels at 4 and 7 p.i.; there were also increased levels of protein compared to uninfected controls at 10 and 14 days p.i. Both IFNβ protein and mRNA levels decreased at 21 days p.i. At 42 days p.i. there was no correlation between SIV RNA levels and either IFNβ mRNA or protein expression (Figure 4 and Table 3).

TNFα response occurs concomitant with type I IFN responses and is one of the first responses to viral infection of macrophages. TFNα is induced, like IFNβ, through the intracellular RNA helicase, RIG-I [52]–[55]. TNFα mRNA in the brain was increased at 4 days p.i. (Figure 3), reached the highest level at 10 days p.i., and then decreased to pre-infection levels by 14 days p.i. This pattern of change during acute SIV infection in the brain paralleled that observed for IFNβ mRNA. At 42 days p.i., TNFα mRNA levels fell into two groups: 3/9 macaques had levels of induction greater than 20-fold, while 6/9 had levels of induction 6-fold or lower (Figure 3C). Unlike IFNβ mRNA levels at 42 days p.i, TNFα mRNA correlated with SIV RNA levels (r = 0.86, p = 0.003).

### Relative Expression of C/EBPβ-2 and C/EBPβ-3 in Brain at 42 Days P.I

We previously demonstrated that there is induction of C/EBPβ-3—the dominant-negative isoform of C/EBPβ—in the brain of SIV-infected macaques at 7, 10 and 21 days p.i., and that the level of C/EBPβ-3 correlates with the suppression of SIV RNA [14], [15]. Here we examined the levels of C/EBPβ-2 and C/EBPβ-3 in the brain of the SIV-infected macaques euthanized at 42 days p.i. to determine whether there was a correlation between the ratio of C/EBPβ-3:C/EBPβ-2 and the level of expression of viral RNA.

Since there are no antibodies that distinguish the C/EBPβ-2 and C/EBPβ-3 isoforms, quantitative Western blot analyses were performed on brain homogenates from macaques euthanized at 42 days p.i. and the ratio of C/EBPβ-3:C/EBPβ-2 was measured (Table 4). There was an inverse correlation (r = −0.78, p = 0.02) between the ratio of C/EBPβ-3:C/EBPβ-2 and the level of SIV RNA in the brain. There was also an inverse correlation with IL6 mRNA levels (r = −0.67, p = 0.05). We observed previously that higher ratios of C/EBPβ-3:C/EBPβ-2 correlated with reduction of SIV replication during acute infection and these results supports a strong role for the induction of C/EBPβ-3 by IFNβ and potentially other IFNβ pathways that control SIV replication and cytokines.

**Table 4:** SIV RNA and Ratio of C/EBPβ-3/C/EBPβ-2 in SIV-infected Macaque Brain at 42 Days P.I.

Animal	SIV RNA copy equiv./ug RNA Brain	C/EBPβ-3/C/EBPβ-2
PQw2	49	1.27
PLc2	2,169	1.92
PLi2	10,048	0.76
PWf2	38,600	0.96
PEe2	202,067	0.61
PGe2	238,300	0.71
PFc2	2,104,000	0.80
PRd2	16,226,667	0.63
PLb2	26,876,667	0.49

### Cytokines mRNA Levels in the Brain

**Figure 5:**
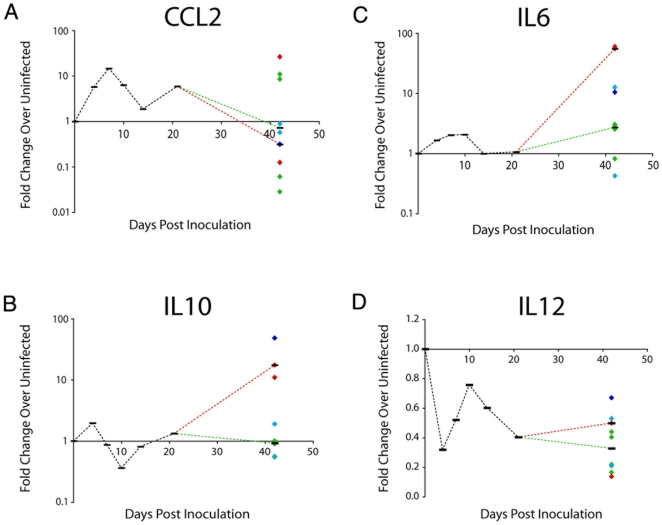
CCL2, IL6, IL10, and IL12 mRNA in brain of SIV-infected macaques. mRNA was isolated form brain of uninfected and SIV-infected macaques at terminal time points (4, 7, 10, 14, 21, or 42 days after inoculation). mRNA levels for A) CCL2; B) IL6; C) IL10; and D) IL12 was measured by quantitative RT-PCR and mRNA levels in the SIV-infected are represented as fold change over the average of the uninfected values, calculated by ΔΔCt. Medians (black bars) for each experimental group are indicated. For the 42-day group, each diamond represents one animal, color-coded according to CNS disease severity (red-severe; blue-moderate; turquoise-mild; green-none). The black bars represent the medians for severe/moderate (red dotted line), and mild/none animals (green dotted line).

CCL2 and IL6 have been correlated with the development of CNS disease in HIV. In addition to CCL2 and IL6 mRNA quantification, we measured mRNA of IL10 and IL12, whose secretion defines differently activated subsets of macrophages (Figure 5) [56]. CCL2 mRNA increased at 4 days p.i., peaked at 7 days p.i. and decreased at 10 and 14 days p.i. At 21 days p.i. CCL2 levels increased in parallel with SIV RNA (Figure 5A). Similarly, IL6 mRNA was induced, consistent with observations in CSF that IL6 changes paralleled those of CCL2. IL6 mRNA levels peaked at 7 days p.i., were at the lowest level of induction at 14 days p.i., and increased at 21 and 42 days p.i. (Figure 5B). IL10 mRNA peaked during acute infection at 4 days p.i. and then declined to uninfected levels by 7–14 days p.i. (Figure 5C). IL10 mRNA levels were elevated again at 21 days p.i. and by 42 days p.i. In contrast to the other cytokines examined, IL12 (p40) mRNA levels were lower than levels in uninfected animals throughout acute infection, never increasing to levels above those in uninfected macaques (Figure 5D). The acute infection levels of IL12 mRNA showed an inverse pattern compared to IL10 mRNA levels. Thus, all the mRNAs examined, except for IL-12, increased during acute infection and then declined to uninfected levels in the brains of the macaques euthanized at 14 days p.i. This appears to represent a coordinated downregulation of the initial inflammatory cytokine responses that are triggered at 4 days p.i. by virus infection in macrophages in brain.

### Expression of Macrophage, Endothelial Cell and Astrocyte Inflammatory Proteins during Acute Infection and Early Disease

To examine the expression of proteins associated with cellular activation or inflammatory responses during acute infection in brain macrophages, astrocytes and endothelial cells, sections of brain (basal ganglia) were stained for CD68 (macrophage activation marker), MHC Class II (macrophage and endothelial cell activation marker), and GFAP (astrocyte activation marker).

**Figure 6:**
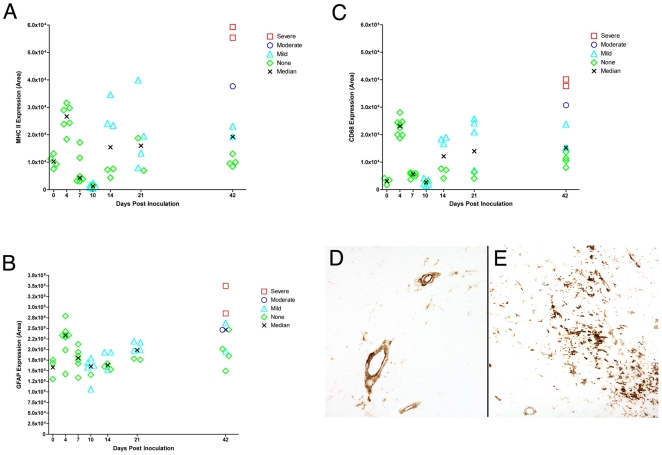
Expression of MHC Class II, CD68 and GFAP in brain of SIV-infected macaques. Quantitative immunohistochemistry was used to measure A) MHC class II; B) CD68; and C) GFAP in brain from SIV-infected macaques at terminal time points (4, 7, 10, 14, 21, or 42 days p.i.). The quantification of each protein is based on the mean of 20 measures on brain from each macaque. D) MHC class II expression in brain at 4 days p.i. E) CD68 expression in brain at 4 days p.i.

At 4 days p.i., MHC Class II and CD68 expression increased in the SIV-infected brain by 2.4- and 7-fold, respectively (Figures 6A and 6B). Expression rapidly returned to uninfected levels at 7 and 10 days p.i. While CD68 is found exclusively in macrophages, MHC Class II is expressed in both macrophages and endothelial cells. At 4 days p.i., MHC Class II was almost exclusively expressed in endothelial cells in the SIV-infected brain (Figure 6), whereas at later time points, it was expressed in endothelial cells but more prominently in macrophages. GFAP expression in astrocytes also increased 1.7-fold at 4 days p.i. (Figure 6C). The expression of all three of these cellular activation proteins decreased by 10 days p.i. and increased expression occurred between 14 and 21 days p.i. At these time points, MHC Class II expression was detected in both macrophages and endothelial cells. At 42 days p.i., the expression level of all three cellular activation proteins correlated with the severity of CNS disease in the brain and with levels of SIV RNA in brain (MHC CLASS II and CD68 r = 0.95, p<0.001; GFAP r = 0.74, p = 0.03) (Table 4).

### CNS Lesions in SIV-Infected Macaques at 42 Days P.I

SIV encephalitis is characterized by numerous perivascular cuffs of epithelioid macrophages and multinucleated giant cells, as well as multifocal nests of activated macrophages and astrocytes in the parenchyma. These lesions are very similar to HIV encephalitis, except that SIV-infected macaque brains often contains more lymphocytes; this is probably due to the fact that macaques are euthanized when they exhibit any clinical signs of disease, so the CNS is likely examined at an earlier stage of CNS disease progression [57].

Approximately 55% (5/9) of macaques euthanized at 42 days p.i. developed neurological disease: 2 macaques were classified pathologically with mild CNS lesions, 1 with moderate CNS lesions, and 2 with severe CNS lesions. In our previous studies in the SIV macaque model, we found that 90% (26/29) of macaques euthanized at 84 days p.i., developed neurological disease and 41% were classified as severe CNS lesions [58]. For macaques euthanized at 42 days p.i., there was a significant correlation between SIV RNA levels and the severity of CNS disease (r = 0.95; p<0.001). The strong correlation between viral RNA levels in the brain and the severity of CNS lesions has been reported previously in this model in SIV-infected macaques euthanized at 84 days p.i., but had not been observed as early as 42 days p.i. [43], [58]. At 42 days p.i., IL6 and IL10 mRNA levels correlated with CNS lesion severity (r = 0.71, p = 0.04 and r = 0.69, p = 0.05, respectively).

## Discussion

While HIV and SIV infect the brain during acute infection, there is no evidence of cognitive alterations or ongoing inflammatory changes in the brain during this early CNS infection. Other studies have demonstrated SIV infection in the brain during early infection, as well as the induction of innate immune responses; however, there are no HIV or SIV studies that have examined all of these viral and cellular changes in the same macaques on a longitudinal basis throughout acute infection [33], [59]. This is due, in part, to the variable course of disease in both HIV infected individuals as well as experimentally SIV-infected macaques that take years to develop AIDS with only a subset of humans or macaques developing CNS disease. In this study we have used our consistent and accelerated SIV model, in which all infected macaques develop AIDS and 90% develop mild-severe CNS disease by 3 months p.i.; the consistent CNS disease development makes this model ideal for the study of CNS lesion development and associated events in the brain. In this study a total of 55 SIV-infected macaques were euthanized at 4, 7, 10, 14, 21 and 42 days p.i. to examine the viral and cellular changes that occur from acute infection at 4–10 days p.i. to the early development of disease between 21 and 42 days p.i.

These studies demonstrate for the first time that a transient but effective innate immune response in brain effects coordinated control of both virus replication and the pro-inflammatory cytokines produced in response to acute infection. We demonstrate that SIV replication occurs in the brain as early as 4 days p.i., predominantly in perivascular macrophages. Virus infection in the brain at 4 days p.i. was accompanied by IFNβ and TNFα responses; these antiviral responses waned rapidly by 14 days and there was control of virus replication during this period. IFNβ and MxA mRNAs were upregulated in both the CD14+ and CD11b+ macrophage populations from the brain, while virus replication was predominantly in the CD14+ macrophages, suggesting that innate immune responses are more widespread than the SIV infection in these cells. Thus, both SIV infection and the accompanying innate immune response in brain and in macrophages enriched from brain are detectable as early as 4 days p.i.—at the same time that virus replication is detected in the peripheral blood.

**Figure 7:**
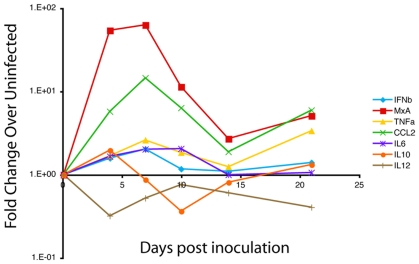
Coordinated expression of innate immune genes and cytokines in brain of SIV-infected macaques during acute infection in brain. A schematic of median values of mRNA levels for all cytokines measured by RT-PCR in brain tissue from uninfected and SIV-infected macaques euthanized at different time points (4, 7, 10, 14 and 21 days p.i.).

In addition, the induction of the cytokines IL6, IL10, and the chemokine CCL2 occurred during acute infection (Figure 7). CCL2 is produced in astrocytes in response to HIV and SIV infection of the CNS [35], [60], [61]. CCL2 secretion in the brain is thought to create a gradient that recruits peripheral blood monocytes and activated and infected lymphocytes into the brain [62], [63]. In addition, production of CCL2 by astrocytes has been demonstrated *in vitro* to have neuroprotective effects in astrocytes and neurons by inhibiting apoptosis [64].

We also observed induction of the dominant-negative isoform of C/EBPβ, C/EBPβ-3, which is a key mediator in this cytokine cascade. The C/CAAT family protein C/EBPβ is important in regulating the expression of cytokines and has been implicated in anti-inflammatory control by IFNβ [26], [29], [30], [40], [65], [66]. There are multiple protein isoforms of C/EBPβ and these differ in structure and function. The major isoform translated from the second AUG in the C/EBPβ mRNA (referred to LAP in rodents and C/EBPβ-2 in humans) contains the transcriptional activation domain as well as the DNA binding domain and activates transcription. The isoform that is translated from the third AUG in the C/EBPβ mRNA (called LIP in rodents and C/EBPβ-3 in humans) contains only the DNA binding domain and functions in the presence of LAP or C/EBPβ-2 as a dominant-negative transcriptional repressor [16], [67]. The ratio of the two isoforms is important in regulating transcription of a number of cytokine genes, including CCL2, IL-6, IL-10 and TNF-α. The C/EBPβ-3:C/EBPβ-2 ratio also regulates SIV transcription *in vitro* in primary macrophages as well as SIV replication in brain (in which macrophages/microglia are the primary productively infected cells) [14], [25]. In this previous study, the level of C/EBPβ-3 in brain of SIV-infected macaques increased from 7–21 days and this correlated with the reduction of SIV mRNA in brain, as well as the level of acetylation of histone H4 downstream from the SIV transcriptional start site. Unlike C/EBPβ-2, C/EBPβ-3 does not recruit histone acetylases to the promoter and the promoter is deacetylated when C/EBPβ-3 is present in the promoter and transcription is suppressed [14], [15], [25].

Both IL-10 and IFNβ have been demonstrated to induce the expression of C/EBPβ-3, contributing to the induction of this isoform of C/EBPβ in the brain during acute infection [23]. IFNβ and IL10 induction of C/EBPβ-3 plays an important role in the anti-inflammatory control for these two cytokines in macrophages and it appears that the level of C/EBPβ-3 correlate with expression of virus during acute infection, as well as the level of virus replication at 42 days p.i. Thus, in this study we have demonstrated that a cellular transcription factor that is induced by IFNβ and IL-10 is important in the brain for regulating virus replication and the expression of pro-inflammatory cytokines that are strongly linked to CNS inflammation and HIV-associated CNS disease. We have now demonstrated that C/EBPβ-3 levels correlate with not only virus replication in the brain, but the severity of CNS lesions and expression of inflammatory proteins in macrophages, endothelial cells and astrocytes, suggesting that this is an important pathway for controlling the progression of HIV and SIV CNS damage and disease processes.

An exception to the observed general pattern of cytokine induction was expression of the pro-inflammatory cytokine IL12, which was markedly suppressed during all stages of infection. A recent cohort study demonstrated that IL12 expression was deficient in chronically infected HIV individuals, although IL12 was induced in the periphery during acute infection [68]. It is clear from our studies that the inflammatory IL12 response during acute phase is different between the periphery and the CNS, with repression of IL12 observed in brain at all time points in our model. The function of IL12 in peripheral infection is thought to include stimulation of CD4+ help in the CTL process. [69] This role is unlikely to be as important in the brain during acute infection, which is marked by low numbers of brain lymphocytes and low proportions of CTLs and NKs [70].

The transient expression of CD68 in macrophages, MHC Class II in endothelial cells and GFAP in astrocytes at 4 days p.i. demonstrates that cellular pro-inflammatory responses are triggered during acute infection and, like the cytokine and chemokine responses, rapidly return at 7–10 days p.i. to levels observed in uninfected macaques. This provides evidence at the cellular level that the coordinated regulation of pro-inflammatory genes occurs in endothelial cells, perivascular macrophages and astrocytes. The decreased expression of MHC Class II expression on endothelial cells would be expected to decrease the entry of monocytes and lymphocytes into the CNS and contribute to the control in virus replication observed.

Our studies further indicate the development of two patterns of viral replication in brain as early as 21 days p.i.: one characterized by SIV RNA levels lower than day 10 and 14 p.i. levels and one characterized by higher levels. Indeed at 42 days p.i. the two levels of virus replication strongly correlate with the severity of CNS lesions, as well as CD68, MHC CLASS II and GFAP protein levels in brain. We also observed a correlation between IL6 and IL10 levels and CNS lesion development at 42 days p.i..

The coordinated control of virus replication and inflammation suggests that there are specific—and possibly common—mechanisms in the brain that limit inflammatory processes produced in response to HIV and SIV infection and provides an explanation for the lack of neurological deficits and CNS inflammation during acute HIV infection in the brain. Our data suggest that the level of C/EBPβ-3 in brain plays an important role in regulation, although there are likely additional pathways that are involved in regulating the inflammatory responses. If the regulatory mechanisms that impact both virus replication and pro-inflammatory cascades fail to control virus replication, virus then induces both IFNβ and TNFα; however, at this stage of the infection (by 21 days p.i.) adaptive immune responses have been induced and the presence of the adaptive along with the innate immune responses probably contribute to the inability of the anti-inflammatory mechanisms to regulate either virus replication or infection-induced inflammatory responses.

We have previously demonstrated that treatment with minocycline, an antibiotic that has anti-inflammatory effects in the brain, can impact the inflammatory response to SIV at 21 days p.i. when initiated after acute infection [71]. The data reported here, together with the minocycline studies, suggest that therapeutic intervention with specific drugs that control HIV infection and/or the inflammatory responses in the brain could prevent the cognitive changes and encephalitis that continue to affect HIV-infected individuals in the HAART era. Further, these studies indicate that increases in either virus or CCL2 in the CSF clearly mirror events in the brain and should be monitored in HIV infected individuals and used to initiate either CNS-penetrating antiretrovirals or anti-inflammatory therapy capable of crossing the blood brain barrier.

These studies demonstrate that there are mechanisms in brain that induce coordinated control of both virus replication and the inflammatory cytokines produced in response to infection. The results suggest that the inflammatory responses required to limit virus replication in the brain must also be tightly controlled so as to prevent the development of chronic inflammation that can trigger neuronal damage and cognitive impairment. A loss of this inflammation-limiting capacity may contribute to the progression to encephalitis in late stage disease. The studies reported here provide the first step in identifying these pathways and our model will be used to identify additional cellular signaling pathways that regulate infection and inflammatory changes in the brain.

## Methods and Materials

### Viruses and Animal Studies

Forty-four juvenile pigtailed macaques (*Macaca nemestrina*) were intravenously inoculated as previously described with SIV/DeltaB670 (50 AID_50_) and SIV/17E-Fr (10,000 AID_50_) [43]. CSF and plasma samples were taken on days 7, 10, 14, 21, 28, 35, 43, 56, 70, 77 and 84 for quantitation of viral RNA, and ELISA quantitation of monocyte chemoattractant protein (CCL2) and IL6 [36], [37], [43]. Macaques were euthanized at 4 (6 macaques), 7 (11 macaques), 10 (12 macaques), 14 (6 macaques), 21 (12 macaques) and 42 (9 macaques) days p.i. in accordance with federal guidelines and institutional policies. At euthanasia, macaques were perfused with sterile saline to remove blood from the vasculature prior to freezing or fixing tissues.

All animal studies were approved by the Johns Hopkins University Institutional Animal Care and Use Committee and in accordance with the recommendations of the Weatherall Report. Early endpoints are adopted for this study including aggressive monitoring of bloodwork parameters and humane euthanasia once progressive disease is noted. All animal housing and care is conducted according to the Guide for the Care and Use of Laboratory Animals and the United States Department of Agriculture Animal Welfare Act. All non-human primates receive environmental enrichment including manipulanda, foraging, and opportunity to exhibit species-specific behavior. Animals are pair or group housed when possible.

### Quantitation of SIV Virions in Plasma and CSF

Virus was quantitated in plasma and CSF from 140 µl of plasma and CSF collected longitudinally as well as at the terminal time point. Viral RNA was isolated directly from plasma and CSF using the QIAamp Viral RNA Mini kit (Qiagen), according to the manufacturer's protocol. Quantification of virion-associated RNA was performed by real-time RT-PCR as previously described [15].

### Quantitation of Viral and Cellular Genes in Brain Tissue

Total RNA was isolated from 50 mg of brain tissue (basal ganglia and parietal cortex) by use of the RNeasy kit (Qiagen), and treated with two units of Turbo DNase (Ambion) for 30 minutes at 37°C. One microgram of purified RNA was analyzed by real-time RT-PCR using specific primers and probes for SIV gag [15] and each of the studied cytokines (Table S1). PCR reactions were performed in a Chromo4 thermocycler (Biorad) using a Multiplex PCR Mix (Qiagen). Cellular mRNA levels were normalized by 18S ribosomal RNA levels. Quantitation of gene expression was performed using the ΔΔ Ct method [72].

### Quantitation of IFN-ß and C/EBP-ß Proteins

Western blot analysis was performed on lysed punches of brain tissue (snap-frozen). Briefly, 50 mg of brain tissue was homogenized in RIPA buffer containing protease inhibitors (Sigma). Proteins (40–80 µg) were separated on 4–12% SDS-polyacrylamide gels and transferred onto polyvinylidene difluoride membranes (PVFD) membranes. Blots were blocked with 0.5% fetal bovine serum and probed for the respective proteins with specific primary antibodies, using a Snap ID apparatus (Millipore). C/EBPβ (C-19) and GAPDH antibodies were purchased from Santa Cruz. Human IFNβ antibody was purchased from PBL InterferonSource (Piscataway, NJ). After incubation with fluorescence-conjugated secondary antibodies (GE Healthcare; Invitrogen), the membranes were visualized on a Typhoon 9400 scanner (GE Healthcare). Band intensities were measured and analyzed using ImageQuant software (GE Healthcare). For the IFNβ western blots, equal protein loading was confirmed by comparison with the intensity of GAPDH.

### Isolation of CD14+ and CD14−/CD11b+ Brain Cells

Microglial cells from the subcortical white matter from six macaques sacrificed at 4 days p.i. were isolated as previously described [45]. CD14+ cells were selected with specific magnetic Dynabeads (Invitrogen) according to the manufacturer's protocol. After three washes with wash buffer (2% BSA in PBS), the unselected portion was promptly incubated with CD11b+ Dynabeads (Invitrogen). Both CD14+ and CD14−/CD11b+ cell populations were snap-frozen for future RNA isolation.

### Quantitative Immunohistochemical Analysis

Our methods for quantitative immunohistochemical analysis of CD68, MHC CLASS II and GFAP have been described previously [15], [36], [43], [70].

### Pathological Assessment

All tissues were examined microscopically by two pathologists (CZ, JM). Sections of frontal and parietal cortex, basal ganglia, thalamus, midbrain, cerebellum and brain stem were examined microscopically and scored independently as mild, moderate, or severe and were each given numerical scores of 1 (mild), 2 (moderate), or 3 (severe) by using a semiquantitative system as described [43].

### Statistical Analysis

A non-parametric method of comparison (Wilcoxon rank-sum test) was used for comparisons between SIV encephalitis severity groups of macaques (i.e., none and mild versus moderate and severe groups). T-tests were not performed since many variables required mathematical transformations (e.g. Log 10) to meet normality requirements. Expression of MHC II, CD68, GFAP and gp41 in the brain were quantitated using 20 separate measures on each tissue sample; the mean was used for analyses. Spearman's rank correlation test was used to determine the degree of correlation between each measure. Spearman's is an analogous non-parametric to be used in place of Pearson's estimate if either variable under consideration is found to be highly skewed. Non-parametric methods are considered to be conservative; therefore statistically significant results found when using non-parametric methods are assumed to imply a lower bound for the p-value. All statistical tests were performed as two-sided tests.

## Supporting Information

Table S1 Primers and probes for SIV gag and cytokines. Primers and probes used for real-time RT-PCR analysis of SIV gag and each of the studied cytokines.
(0.39 MB TIF)
