# Supplementary figures and images for: Coordinated Regulation of SIV Replication and Immune Responses in the CNS
Source: PLoS One. 2009 Dec 17;4(12):e8129. doi: 10.1371/journal.pone.0008129 (PMC2790080; doi:10.1371/journal.pone.0008129)

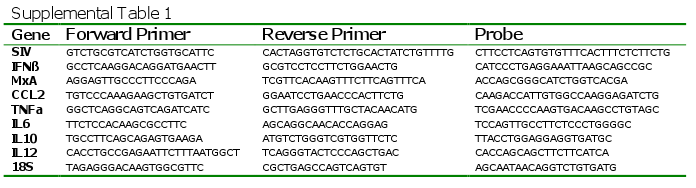

Supplement: Table S1 — Primers and probes for SIV gag and cytokines. Primers and probes used for real-time RT-PCR analysis of SIV gag and each of the studied cytokines. (0.39 MB TIF) [file pone.0008129.s001.tif]
